# The effect of preemptive intravenous paracetamol-mannitol on postoperative analgesia and quality of recovery in elderly patients undergoing total hip arthroplasty

**DOI:** 10.1186/s12871-026-03815-x

**Published:** 2026-04-09

**Authors:** Xinyuan Zhou, Jun Jing, Pu Xu, Qianwen Wu, Meiyin Chen

**Affiliations:** 1 Ma’anshan People’s Hospital, Wannan Medical College, Wuhu, Anhui Province 241003 China; 2 Department of Anesthesiology, Ma’anshan People’s Hospital, Ma’anshan, Anhui Province 243000 China

**Keywords:** Paracetamol, Hip Arthroplasty, Elderly Patients, Multimodal Analgesia, Preemptive Analgesia

## Abstract

**Background:**

Total hip arthroplasty (THA) is a common orthopedic surgery for elderly patients, but it is often associated with severe postoperative pain, leading to increased opioid consumption and delayed recovery. Against the backdrop of the increasing popularity of multimodal analgesia strategies, this study aimed to investigate the effects of preemptive intravenous administration of paracetamol-mannitol injection on postoperative analgesia, inflammatory response, and quality of recovery in elderly patients undergoing THA.

**Methods:**

Sixty-six elderly patients scheduled for elective THA were randomly allocated using a random number table into either the experimental group (Group A, receiving paracetamol-mannitol injection 30 min before surgery) or the control group (Group C, receiving an equal volume of normal saline). All patients received a sufentanil-based patient-controlled intravenous analgesia (PCIA) pump postoperatively. The primary endpoint was the Visual Analog Scale (VAS) score at rest 24 h postoperatively. Secondary endpoints included cumulative 24-hour sufentanil consumption, time to the first analgesic request, number of effective PCIA demands, Brugge-mann Comfort Scale (BCS) scores, incidence of adverse reactions, and changes in serum levels of inflammatory markers (IL-1*β*, IL-6, TNF-*α*) and the neuro-stress protein S100*β*.

**Results:**

All 66 enrolled patients completed the study with no dropouts. The baseline characteristics of the two groups were comparable. Compared to Group C, Group A exhibited significantly lower VAS scores at 6 and 24 h postoperatively, a significant reduction in total 24-hour sufentanil consumption and the number of PCIA demands, and a significantly prolonged time to the first analgesic request (*P <* 0.05). Furthermore, Group A exhibited significantly higher comfort scores at 6 h postoperatively (*P <* 0.05). At 24 h postoperatively, there was a significant statistical difference in serum levels of the inflammatory marker TNF-*α* and the neuro-stress indicator S100*β* between the two groups (*P <* 0.05). Moreover, within Group A, there was no significant statistical difference between preoperative and postoperative levels of TNF-*α* and S100*β* (*P >* 0.05). There was no statistically significant difference in the incidence of postoperative adverse reactions between the two groups (*P >* 0.05).

**Conclusion:**

In elderly patients undergoing total hip arthroplasty, preemptive intravenous acetaminophen-mannitol effectively alleviates early postoperative pain, reduces opioid consumption, and enhances patient comfort, possibly by attenuating postoperative inflammation and neurostress responses to improve perioperative recovery; however, the clinical significance of associated changes in inflammatory and neurostress markers within 24 h postoperatively remains to be further elucidated.

**Trial registration:**

The trial was registered on Aug 6, 2025 in the Chinese Clinical Trial Registry(https://www.chictr.org.cn/bin/user Project = 257710),registration number ChiCTR 2,500,107,208 (06/08/2025).

**Supplementary Information:**

The online version contains supplementary material available at 10.1186/s12871-026-03815-x.

## Introduction

With the accelerating pace of global population aging, the burden of age-related degen- erative diseases is increasing. End-stage hip disease, a typical example, is commonly caused by severe osteoarthritis, avascular necrosis of the femoral head, rheumatoid arthritis, and specific types of femoral neck fractures. It often leads to intractable pain and significant functional impairment, severely impacting the quality of life of elderly patients [1, 2]. Total Hip Arthroplasty (THA) is regarded as one of the most successful and mature surgical procedures of the 20th century, benefiting millions of patients worldwide annually [3]. However, THA itself is a major traumatic orthopedic surgery involving extensive soft tissue dissection, acetabular reaming, and femoral prosthesis implantation, frequently resulting in moderate to severe acute postoperative pain, ranking among the most painful of all surgical procedures [4].

Postoperative pain not only causes significant subjective suffering but can also trigger a cascade of adverse pathophysiological responses, which are particularly detrimental to elderly patients with diminished physiological reserves. Firstly, acute pain activates the sympathetic-adrenal system, leading to a massive release of catecholamines, causing tachycardia, hypertension, and increased myocardial oxygen consumption. For elderly patients with comorbid cardiovascular diseases, this significantly increases the perioperative risk of myocardial ischemia, arrhythmias, and even acute heart failure [5, 6]. Secondly, multiple studies have confirmed that pain is an independent high-risk factor for Postoperative Delirium (POD) [7, 8]. The incidence of POD in elderly patients undergoing THA or hip fracture surgery is as high as 30–50% [9, 10], which not only significantly prolongs hospital stays and increases healthcare costs but is also associated with long-term cognitive decline and increased mortality. Furthermore, pain-induced protective immobilization hinders early ambulation, delays the rehabilitation process, and increases the risk of complications such as deep vein thrombosis, pulmonary embolism, and pulmonary infections [11].

In traditional analgesic models, potent opioids (e.g., morphine, fentanyl) delivered via patient-controlled intravenous analgesia (PCIA) provide individualized pain relief. Although opioids are effective in alleviating moderate to severe pain, their extensive adverse effects are more pronounced and dangerous in the elderly population, including nausea and vomiting, excessive sedation, respiratory depression, paralytic ileus, and urinary retention [12]. More importantly, opioid-related central nervous system side effects can increase the risk of POD and long-term postoperative cognitive dysfunction. Due to decreased hepatic and renal function and slowerSignificance of Preemptive Analgesia and Opioid Sparing drug clearance in elderly patients, the half-life of opioids is prolonged and the risk of accumulation is increased, making the balance between effective analgesia and adverse effects more difficult to achieve [13]. In clinical practice, adverse reactions from opioids not only reduce patient satisfaction but also delay ambulation and prolong hospital stays, creating a dilemma of “sacrificing overall recovery quality for pain relief,” which runs counter to the core principles of Enhanced Recovery After Surgery (ERAS) that advocate for “fast, painless, and safe recovery” [14, 15].

In recent years, the Multimodal Analgesia (MMA) strategy has gradually become the internationally recognized best practice. MMA emphasizes the combination of drugs or techniques with different mechanisms of action, such as non-steroidal anti-inflammatory drugs (NSAIDs), paracetamol, gabapentinoids, NMDA receptor antagonists, *α* 2-agonists, and regional nerve blocks, to achieve better analgesic effects through synergistic action while producing an opioid-sparing effect and reducing the incidence of adverse reactions [16]. Within the MMA framework, paracetamol is recommended as a fundamental analgesic by the World Health Organization (WHO) and various national guidelines due to its high safety profile and lack of significant gastrointestinal or cardiovascular side effects [17]. Its intravenous formulation, with its rapid onset, high bioavailability, and low risk of hepatorenal toxicity, plays a crucial role in multimodal analgesia [18]. The novel compound formulation, paracetamol-mannitol injection, exhibits central analgesic effects, but its value in the perioperative period for elderly patients, especially as a preemptive analgesic measure, requires high-quality clinical trial evidence [19]. Although intravenous paracetamol is widely used in multimodal analgesia, its isolated preemptive effect in elderly patients undergoing major orthopedic surgery remains incompletely characterized. Moreover, limited data are available regarding its association with perioperative inflammatory and neuro-stress markers in this high-risk population. Therefore, this randomized controlled trial was designed to evaluate the clinical and exploratory mechanistic impact of a single preoperative intravenous dose of paracetamol in elderly patients undergoing THA.Based on the current literature, we hypothesized that preemptive intravenous administration of paracetamol-mannitol injection would significantly reduce postoperative pain intensity and cumulative opioid consumption, attenuate systemic inflammatory and neurogenic stress responses, and enhance the quality of recovery in elderly patients undergoing total hip arthroplasty (THA), compared with a placebo control.

## Materials and methods

### Study design and ethical approval

This was a single-center, prospective, randomized, placebo-parallel-controlled, superiority clinical trial. The study protocol, implementation, data analysis, and reporting strictly adhered to the Consolidated Standards of Reporting Trials (CONSORT) statement. Prior to initiating any study-related procedures, the complete protocol, informed consent form template, and other relevant documents were submitted to and received formal written approval from the Ethics Committee of Ma’anshan People’s Hospital (Ethics Approval No: 2024-02-(04)). The trial was registered with the Chinese Clinical Trial RegisStry (ChiCTR2500107208) to ensure transparency and traceability(Retrospectively registered).

### Study population

This prospective, randomized, controlled clinical trial was conducted between June 2024 and July 2025 at our hospital. The study protocol was approved by the Ethics Committee of Ma’anshan People’s Hospital, and all participants provided written informed consent before enrollment. A total of 66 elderly patients scheduled for elective unilateral THA were included.

#### Inclusion criteria

1. age ≥ 65 years;
2. Body Mass Index (BMI) between 18.5 and 29.9 kg/m2;
3. American Society of Anesthesiologists (ASA) physical status I-III;
4. undergoing primary THA;
5. no severe cardiac, pulmonary, hepatic, or renal dysfunction;
6. no preoperative cognitive impairment, with a Montreal Cognitive Assessment Basic (MoCA-BC) score ≥ 26（We are using the MoCA scale,which is widely accepted for non-commercial academic research in our region,and it can be used reasonably.);
7. no history of allergy to the study drugs.

#### Exclusion criteria

1. long-term use of opioids or NSAIDs;
2. history of drug or alcohol abuse;
3. presence of severe pain or neuropathic pain preoperatively;
4. inability to understand and use the Visual Analog Scale (VAS) for pain or the PCIA device.

### Blinding

**Fig. 1:**
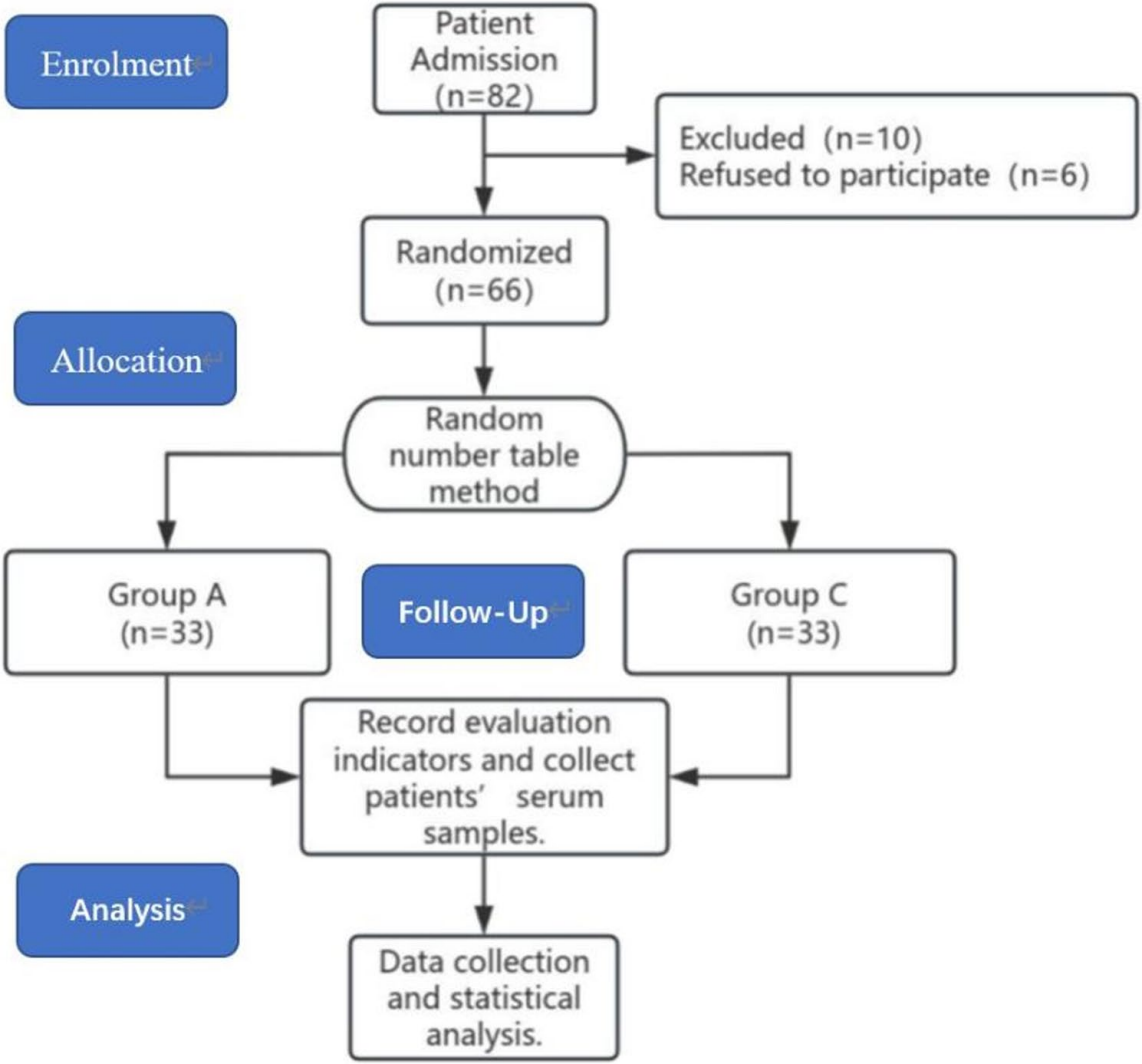
Consolidated Standards of Reporting Trials (CONSORT) flow diagram

The study employed a prospective, randomized, double-blind, placebo-controlled design. Following the CONSORT statement standards, a total of 66 eligible participants were enrolled, with the screening, recruitment, and study flow detailed in Fig. 1. Participants were allocated in a 1:1 ratio to either the experimental group (Group A, *n* = 33) or the control group (Group C, *n* = 33) using a random number table. To achieve allocation concealment, each allocation (”Group A” or ”Group C”) was printed and placed in a sealed, opaque, sequentially numbered envelope. A designated pharmacist, not involved in clinical management, opened the next envelope in sequence only after a patient was confirmed eligible and had signed the informed consent form. This process ensured that all parties, including investigators and patients, could not predict or influence group assignment before enrollment. The study drug was managed by the designated pharmacist. Group A received paracetamol-mannitol injection, while Group C received an equal volume of 0.9% sodium chloride injection as a placeb(The paracetamol-mannitol solution and saline were visually indistinguishable in color and transparency, with no discernible odor or viscosity differences.). Throughout the study, patients, anesthesiologists and surgeons responsible for perioperative management, research personnel conducting postoperative assessments and data collection, and statisticians analyzing the data before unblinding were all blinded to group allocation.

### Interventions

Upon entering the operating room, all patients had a peripheral intravenous line established in the upper limb and were connected to standard vital signs monitoring. According to the randomization result, when the anesthesiologist confirmed that induction was imminent and the surgical incision was expected within 30 min, the circulating nurse administered the study drug infusion. Patients in Group A received an intravenous infusion of pre-prepared paracetamol-mannitol injection (50 mL), containing 500 mg of paracetamol and 1.925 g of mannitol(as an excipient), via the established intravenous access, while patients in Group C received an equal volume of 0.9% sodium chloride solution. Both solutions were colorless and clear and were placed in identical 50 mL syringes labeled only with the patient’s unique random number and the words “Study Medication,” without any information identifying the drug components. The infusion of both solutions was completed at a constant rate of 150–200 mL/h using a standard infusion pump. This timing and infusion rate were chosen to comply with the manufacturer’s recommended administration method and to ensure that the paracetamol-mannitol injection had crossed the blood-brain barrier and reached an effective analgesic concentration in the central nervous system by the time the surgical trauma began.

### Anesthesia and standardized perioperative management

All patients were instructed to follow standard ERAS fasting guidelines: fasting from solid foods (including milk) for 6–8 h and from clear liquids (e.g., water, pulp-free juice) for 2 h before surgery. Upon arrival in the operating room, a peripheral intravenous line (18G or larger) was immediately established in the contralateral (non-operative) upper limb and connected to an infusion set. Standardized multiparameter monitoring was implemented, including electrocardiogram (ECG), pulse oximetry (*SpO*_2_), non-invasive blood pressure (NIBP), Bispectral Index (BIS), and temperature. After confirming the proper functioning of all monitoring equipment, anesthesia induction commenced. Patients were pre-oxygenated with 100% oxygen (flow rate 6–8 L/min) via a face mask for at least 3 min. A standardized general anesthesia induction was then performed using sequential intravenous administration of etomidate (0.15–0.2 mg/kg), sufentanil (0.2 *µ*g/kg), and cisatracurium (0.15 mg/kg) to facilitate endotracheal intubation. After confirming correct tube placement by aus- cultating clear and symmetrical breath sounds, observing good chest wall movement, and detecting a typical end-tidal carbon dioxide waveform, the tube was secured, and the patient was connected to the anesthesia machine for volume-controlled ventilation (VCV). Initial ventilator settings were: tidal volume 6–8 mL/kg (based on ideal body weight), respiratory rate 10–14 breaths/min, I:E ratio 1:2, and an initial *FiO*_2_ of 100%, which was gradually reduced as tolerated while maintaining *SpO*_2_ above 98%. Anesthesia was maintained using a balanced “inhalational-intravenous” technique to leverage synergistic effects and reduce individual drug doses, thereby minimizing side effects. The regimen consisted of continuous sevoflurane inhalation, with the endtidal concentration maintained between 0.8 and 1.5% (approximately 1.0–2.0% inspired concentration) based on BIS values and hemodynamic responses, and a continuous intravenous infusion of propofol (1–2 mg/kg/h) and remifentanil (0.1–0.3 *µ*g/kg/min). The infusion rates of all anesthetic agents were finely adjusted in real-time by the anesthesiologist according to the patient’s individual response. The primary goal for anesthetic depth was to maintain the BIS value within the ideal range of 45–60 to ensure adequate sedation and amnesia, prevent intraoperative awareness, and avoid hemodynamic depression and delayed emergence from excessive anesthesia. Respiratory function was closely monitored, with the respiratory rate adjusted to maintain end-tidal carbon dioxide partial pressure (*EtCO*_2_) strictly within the physiological range of 35–45 mmHg.

Goal-directed hemodynamic management was implemented with proactive interventions based on predefined thresholds to maintain circulatory stability: if heart rate was ≥ 100 bpm for *>* 60 s, intravenous esmolol was administered; if heart rate was *<* 50 bpm, intravenous atropine was given. If SBP was ≥ 180 mmHg, intravenous nicardipine was administered; if SBP was ≤ 80 mmHg, intravenous phenylephrine was given. To ensure a smooth emergence and a seamless transition from intraoperative to postoperative analgesia, sevoflurane was discontinued approximately 30 min before the end of surgery, and the propofol infusion was stopped 15 min before the end. The remifentanil infusion was stopped as the surgeon began closing the final skin layer. To bridge the potential analgesic gap after remifentanil discontinuation, a loading dose of sufentanil 0.1 *µ*g/kg was administered 5–10 min before skin closure to initiate postoperative analgesia. At the end of the procedure, once spontaneous breathing returned, neostigmine and atropine were administered to reverse residual muscle relaxation.

### Postoperative management and analgesic protocol

After surgery, all patients were safely transferred to the Post-Anesthesia Care Unit (PACU) for one-on-one monitoring. In the PACU, ECG, NIBP, *SpO*_2_, and respiratory rate were continuously monitored. The endotracheal tube was extubated only after the patient met all of the following criteria: fully conscious, able to open eyes and lift head for *>* 5 s on command, regular and sufficient spontaneous breathing (tidal volume *> 5* mL/kg), and good recovery of protective reflexes (e.g., cough, swallow). Patients continued to receive oxygen via a face mask and were observed for at least 30 min post-extubation. Discharge criteria from the PACU were: stable vital signs (heart rate and blood pressure within 20% of baseline), no significant nausea or vomiting, initial pain control achieved (resting VAS ≤ 3), and a modified Aldrete score ≥ 9. Upon return to the orthopedic ward, all patients were connected to a pre-configured PCIA pump. To ensure a consistent analgesic background between groups,

the PCIA formulation and settings were standardized: a solution of sufentanil 2 *µ*g/kg diluted to 100 mL with 0.9% sodium chloride. PCIA parameters were set as follows: background infusion rate of 2 mL/h, single bolus dose of 1 mL, and a lockout interval of 15 min. Patients self-administered a bolus when their pain level warrante it (e.g., VAS *>* 3). If a patient’s resting VAS score remained ≥ 4 despite PCIA use, or if the pain was reported as unbearable, a rescue analgesia protocol was initiated: intravenous infusion of flurbiprofen axetil 50 mg. To systematically manage potential PCIA-related side effects, all patients received routine prophylactic antiemetics (e.g., intravenous ondansetron 4 mg at the end of surgery). Postoperatively, researchers monitored and recorded adverse events, which were managed promptly according to hospital protocols.

### Outcome measures and data collection

This study employed a multidimensional set of outcome measures covering postoperative pain control, opioid consumption, physiological and inflammatory stress responses, subjective patient recovery, and overall safety. Postoperative Pain Intensity (Primary Outcome): Assessed using the Visual Analog Scale (VAS, 0–10, where 0 = no pain, 10 = worst imaginable pain) at PACU (T1), and at 6 h (T2), 24 h (T3), and 48 h (T4) postoperatively. Cumulative 24-hour Sufentanil Consumption: The total dose of sufentanil (*µ*g) consumed from the start of PCIA connection until 24 h post-surgery was precisely recorded from the PCIA pump’s electronic log. Time to First Analgesic Request: Defined as the interval (in minutes) from the start of PCIA connection on the ward to the patient’s first effective (i.e., not during a lockout period) press of the PCIA button. This reflects the duration of the baseline analgesic regimen’s effect. Number of Effective PCIA Demands: The total number of patient-initiated, effective presses within the first 24 h. Bruggemann Comfort Scale (BCS): A 5-item tool assessing pain, nausea, fatigue, anxiety, and physical discomfort, with each item scored from 0 to 4. The total score ranges from 0 to 20, with higher scores indicating better overall comfort. Assessed at baseline (T0), T1, T2, T3, and T4. Adverse Events: The incidence of the following events was monitored and recorded for 48 h postoperatively: nausea and vomiting, excessive sedation/drowsiness (Ramsay sedation score ≥ 4), postoperative delirium (POD) (screened twice daily using the Confusion Assessment Method [CAM]), respiratory depression (respiratory rate 10 breaths/min or *SpO*_2_ *<*90% on room air for *>* 60 s), pruritus, and other adverse events such as cardiovascular events or fever (temperature ≥ 37.3^*◦*^*C*). Serum Biomarkers: Blood samples were collected one day before surgery (T0, baseline) and 24 h postoperatively (T3, peak stress response). 5 mL of venous blood was drawn into serum separator tubes, allowed to clot, and centrifuged at 3000 rpm for 10 min. The serum was then aliquoted and stored at -80^*◦*^*C* until batch analysis. Serum concentrations of Interleukin−1*β* (IL−1*β*), Interleukin−6 (IL−6), Tumor Necrosis Factor-*α* (TNF-*α*), and S100*β* protein were measured using enzyme-linked immunosorbent assay (ELISA) kits according to the manufacturer’s instructions.

### Statistical analysis

The sample size was estimated based on a pilot study of 10 patients, which showed mean Visual Analog Scale (VAS) scores at 6 h postoperatively of 3.0 ± 1.1 in the intervention group and 3.8 ± 0.7 in the control group. The expected between-group effect size (ΔVAS) was 0.8, and the standard deviation (SD) of VAS scores was referenced from the pilot study data (SD = 1.1 in the intervention group and SD = 0.7 in the control group).With *α* = 0.05 and *β* = 0.1 (90% power), a minimum of 28 participants per group was calculated to be necessary to detect this difference.The reference study for sample size estimation is the aforementioned pilot study of 10 patients (based on our internal pilot study data.). Accounting for a potential dropout rate of 15%, the final sample size was set at 33 per group. Statistical evaluation was performed using the SPSS 25.0 software. The Kolmogorov- Smirnov test was used to assess the normality of continuous variables. Normally distributed variables are presented as mean ± standard deviation (SD) and compared using one-way ANOVA or independent samples t-test for two-group comparisons. Skewed continuous variables are presented as median and interquartile range (IQR) and compared using the Kruskal-Wallis or Mann-Whitney U test. Categorical variables are presented as counts (percentages) and compared using the Pearson’s chi-square test or Mantel-Haenszel chi-square test for trend. Time-to-first PCIA press was compared using the Mann-Whitney U test. VAS and BCS scores at each time point were compared between groups using the independent samples t-test. Changes in biomarkers were assessed using the paired samples t-test. A two-sided *P <* 0.05 was considered statistically significant.All statistical comparisons in this study were performed using two-tailed tests, with a predefined significance level of α = 0.05.

## Results

### Study flow and patient baseline characteristics

During the recruitment period from June 2024 to July 2025, 82 elderly patients scheduled for unilateral THA were assessed for eligibility. Ten were excluded for not meeting inclusion criteria (*n* = 4 with MoCA-BC ≤ 26; *n* = 4 with long-term opioid use; *n* = 2 with recent respiratory infections), and 6 declined to participate. Ultimately, 66 eligible patients provided informed consent and were randomized into the experimental group (Group A, *n* = 33) or the control group (Group C, *n* = 33). All 66 enrolled patients received their assigned intervention and completed all follow-ups and data collection through 48 h postoperatively, with no dropouts or exclusions from the final analysis. As shown in Table 1, there were no statistically significant differences between the two groups in terms of age, sex, BMI, and ASA physical status (*P >* 0.05 for all). This indicates that randomization was successful in balancing known confounding factors.

**Table 1:** Comparison of baseline characteristics between the two groups

Characteristic	Group A (*n* = 33)[95%CI]	Group C (*n* = 33)[95%CI]	t / χ^2^	*P*
Age (years, $\overline{x}$ ±*s*)	77.48 ± 6.43[75.06,79.90]	78.88 ± 6.38[76.49,81.27]	-0.88	0.38^a^
Sex [n (%)]				
Male	9 (27.27)[12.95,46.78]	6 (18.18)[6.94,36.55]	0.35	0.56^b^
Female	24 (72.73)[53.22,87.05]	27 (81.82)[63.45,93.06]		
ASA classification				
Grade Ⅱ	17(51.52)[33.68,69.07]	20(60.61)[42.15,76.85]	0.25	0.62^b^
Grade Ⅲ	16(48.48)[30.93,66.32]	13(39.39)[23.15,57.85]		
BMI ( $\overline{x}$± *s*, kg/m^2^)	25.02 ± 1.16[24.62,25.42]	24.49 ± 1.50[23.97,25.01]	1.60	0.12

Data are presented as mean *±* standard deviation or number (n)

*ASA* American Society of Anesthesiologists, *BMI* Body Mass Index

a Analyzed by independent samples t-test

b Analyzed by Pearson’s chi-square test

### Primary endpoint: postoperative pain intensity (VAS scores)

**Fig. 2:**
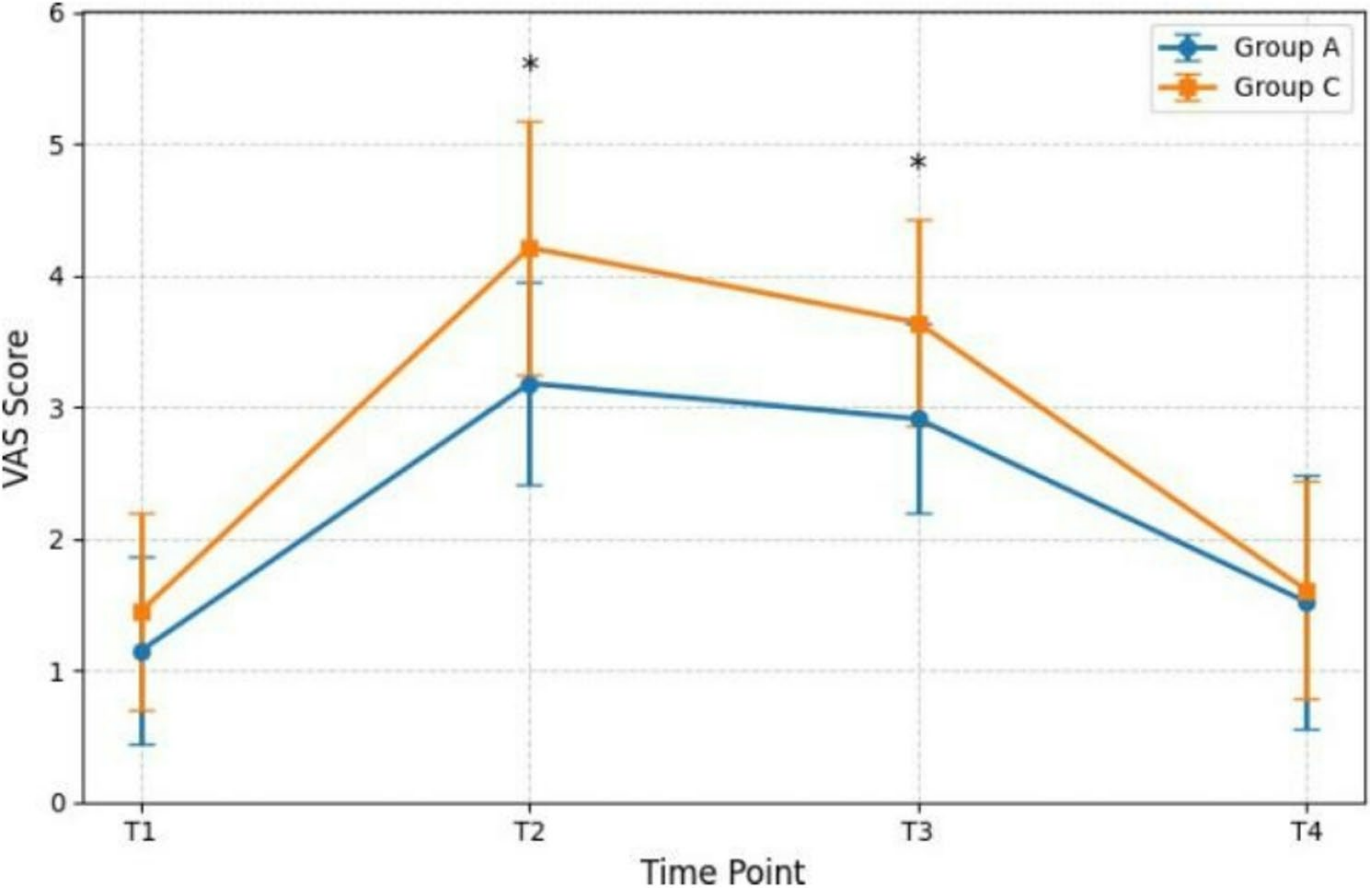
Postoperative Visual Analogue Scale (VAS) scores. T1: at PACU discharge; T2: 6 h postoperatively; T3: 24 h postoperatively; T4: 48 h postoperatively; ^∗^*p <* 0.05 indicates a significant difference between Group A and Group C

As shown in Fig. 2, the primary endpoint of this study was the resting Visual Analog Scale (VAS) score at 24 h postoperatively (T3), with additional comparisons of resting VAS scores at other postoperative time points (T1: Post-Anesthesia Care Unit [PACU]; T2: 6 h; T4: 48 h) for comprehensive analysis. PACU (T1), VAS scores were relatively low in both groups, with no significant difference between them (*P >* 0.05). However, the benefit of preemptive analgesia became evident over time. At 6 h (T2) and 24 h (T3), the periods of expected peak pain intensity, resting VAS scores in Group A were significantly lower than in Group C (*P <* 0.01). By 48 h (T4), as pain naturally subsided and PCIA continued, VAS scores in both groups decreased further to similarly low levels, and the difference was no longer statistically significant (*P >* 0.05).

### Opioid-sparing effect: opioid consumption and analgesic demand

As shown in Table 2, Group A demonstrated a significant opioid-sparing effect. The cumulative sufentanil consumption over the first 24 h was significantly lower in Group A than in Group C (*P <* 0.05). Correspondingly, the number of effective PCIA demands to control breakthrough pain within 24 h was also significantly lower in Group A (*P <* 0.01). Crucially, the time to the first analgesic request (first effective PCIA press) was significantly prolonged in Group A (*P <* 0.05). Furthermore, the number of ineffective presses was also lower in Group A, suggesting more stable pain control. Regarding rescue analgesia, 5 patients in Group C required flurbiprofen axetil compared to only 1 in Group A; however, this difference did not reach statistical significance due to the small number of events.

**Table 2:** Comparison of PCIA usage within 24 h postoperatively

Item	Group A (*n* = 33)[95%CI]	Group C (*n* = 33)[95%CI]	t/U	*P*
Sufentanil consumption (*µ*g)	56.91 ± 3.64[55.62,58.20]	59.67 ± 5.32[57.78,61.56]	-2.46	0.02^a^
Number of PCIA demands	4.09 ± 2.26[3.29,4.89]	6.85 ± 2.88[5.83,7.87]	-4.33	*<* 0.001^a^
Time to first PCIA request (min)	130 (112–160)[126,136]	112 (99–140)[111,122]	712.0	0.03^b^

Data are presented as mean *±* standard deviation or median (interquartile range, IQR)

*PCIA* Patient-Controlled Intravenous Analgesia

a Analyzed by independent samples t-test

b Analyzed by Mann-Whitney U test

### Safety and tolerability: incidence of adverse reactions

As detailed in Table 3, the overall incidence of most common adverse events within 48 h postoperatively was similar between the two groups. There were no statistically significant differences in the rates of nausea, vomiting, dizziness/somnolence (Ramsay score ≥ 4), respiratory distress, postoperative fever, agitation, or POD (*P* > 0.05 for all). A noteworthy trend was observed in the incidence of POD. Only one patient (3.0%) in Group A was diagnosed with POD via the CAM, compared to four patients (12.1%) in Group C. Although the incidence of POD was numerically lower in the paracetamol group (1 vs. 4 cases), this difference did not reach statistical significance (*P* = 0.35).Furthermore, when monitoring the incidence of adverse reactions during postoperative follow-up, no significant changes in renal or liver function indicators were observed in either group compared with the preoperative baseline, and electrolyte levels remained within the clinically acceptable range throughout the entire perioperative period.

**Table 3:** Comparison of postoperative adverse reactions [n(%)]

Adverse Reaction [*n*(%)]	Group A (*n* = 33)[95%CI]	Group C (*n* = 33)[95%CI]	χ^2^	*P* value
Postoperative fever	3(9.10)[3.14,23.57]	5(15.15)[6.65,30.92]	0.14	0.71
Dyspnea	2(6.10)[1.68,19.61]	3(9.10)[3.14,23.57]	0.00	1.0
Agitation	1(3.03)[0.54,15.32]	4(12.12)[4.82,27.33]	0.87	0.35
Dizziness or somnolence	5(15.15)[6.65,30.92]	3(9.10)[3.14,23.57]	0.14	0.71
Nausea or vomiting	4(12.12)[4.82,27.33]	3(9.10)[3.14,23.57]	0.00	1.00
Postoperative delirium	1(3.03)[0.54,15.32]	4(12.12)[4.82,27.33]	0.87	0.35

Data are presented as number (percentage). Analyzed by Pearson’s chi-square test or Fisher’s exact test where appropriate

### Patient subjective experience: comfort scores (BCS)

**Fig. 3:**
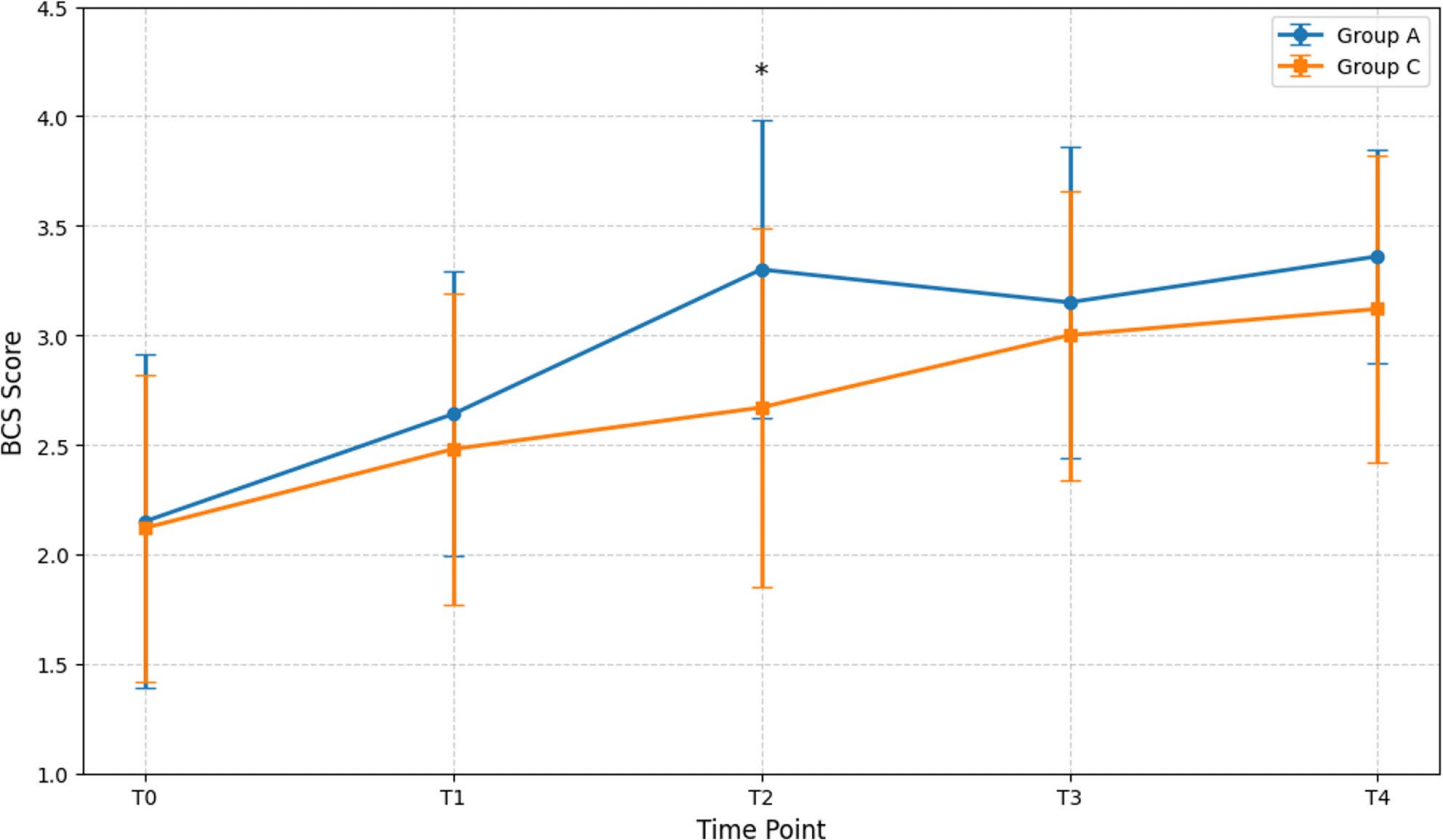
Bruggemann Comfort Scale (BCS) scores under five time points. T0: preoperative; T1-PACU: immediately upon return to the ward; T2: 6 h postoperatively; T3: 24 h postoperatively; T4: 48 h postoperatively. ∗*p <* 0.05 indicates a significant difference between Group A and Group C

Patient comfort was significantly improved in Group A during the early postoperative period, as shown in Fig. 3. Specifically, at 6 h postoperatively (T2), the BCS score in Group A was significantly higher than in Group C (*P <* 0.01). This time point corresponds with the greatest difference in VAS scores, clearly indicating that superior pain control translated directly into a better overall patient experience, encompassing less pain, nausea, fatigue, and anxiety. At other time points, the differences in BCS scores were not statistically significant (*P >* 0.05), which aligns with the trend in VAS scores.

### Mechanistic exploration: inflammatory cytokines and neuro-stress proteins

**Fig. 4:**
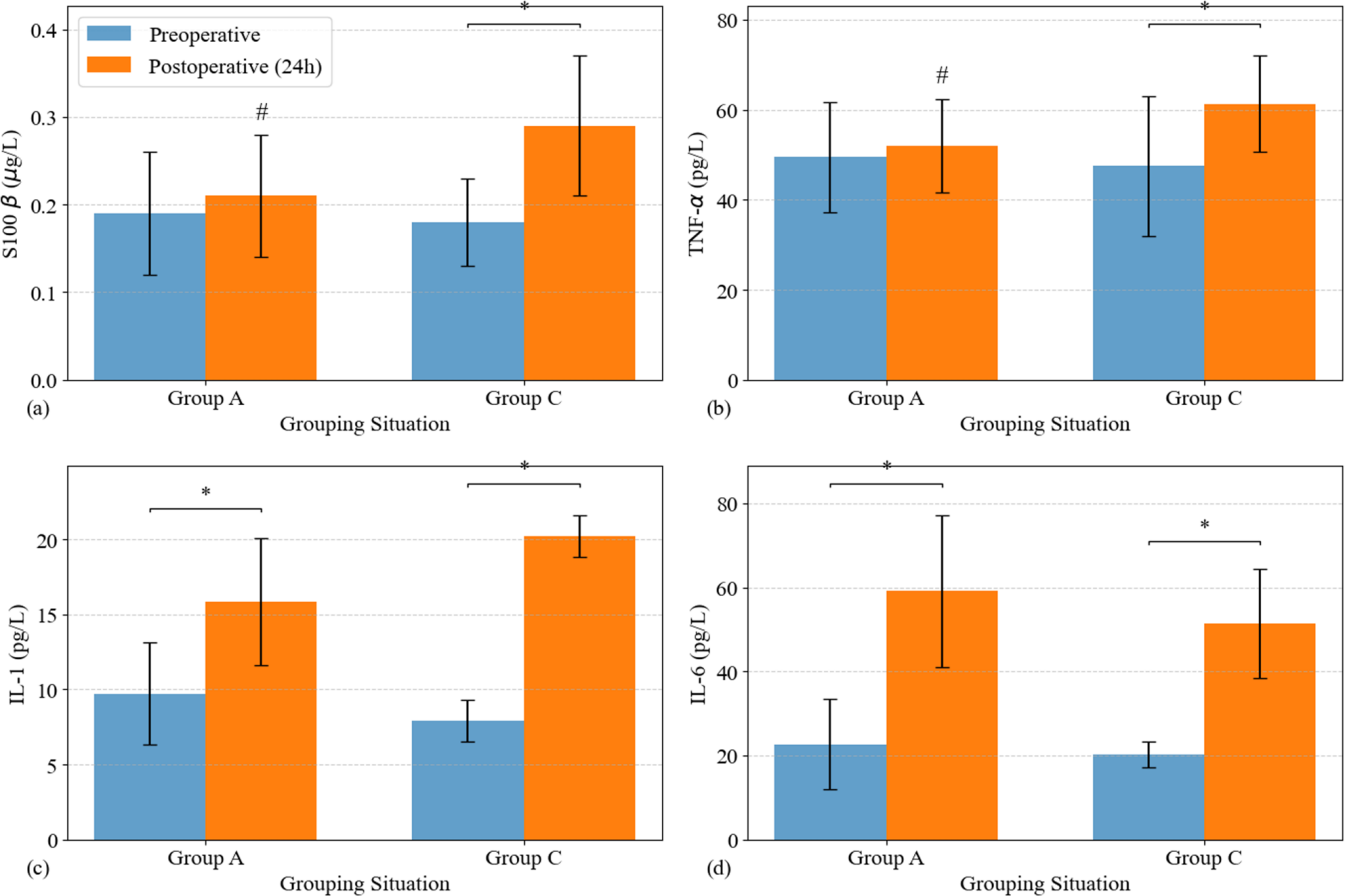
Inflammatory mediators (S100*β*, TNF-*α*, IL-1*β*, IL-6) before surgery and 24 h after surgery, ∗*p <* 0.05, comparison within the same group before surgery and 24 h after surgery. #*p <* 0.05 indicates a significant difference between Group A and Group C at 24 h postoperatively

As shown in Fig. 4 at 24 h postoperatively, serum levels of the major pro-inflammatory cytokines IL-1*β* and IL-6 were significantly elevated from their preoperative baselines in both groups (all *P <* 0.01), consistent with the typical inflammatory response to major surgery. There were no significant between-group differences in the postoperative levels or the magnitude of increase for IL-1*β* and IL-6 (*P >* 0.05). However, a significant difference was observed in the response patterns of two other key markers: TNF-*α* and the neuro-stress protein S100*β*. In Group C, both TNF-*α* and S100*β* levels were significantly elevated at 24 h post-op compared to baseline (*P <* 0.01), reflecting a strong, unmitigated systemic inflammatory and central nervous system stress response. In stark contrast, in Group A, the postoperative levels of TNF-*α* and S100*β* showed no significant difference compared to their respective preoperative baselines (*P >* 0.05). More importantly, the absolute postoperative levels and the magnitude of increase from baseline for TNF-*α* and S100*β* in Group A were significantly lower than those in Group C (*P <* 0.05). This result strongly suggests that preemptive administration of paracetamol-mannitol significantly inhibits specific trauma-induced inflammatory pathways (potentially mediated by TNF-*α*) and the central nervous system stress response (marked by S100*β* release), which may be the physiological basis for its superior analgesic effect and improved patient experience.

## Discussion

THA, while an effective treatment for end-stage hip disease, presents a major challenge in postoperative pain management. In elderly patients, pain not only causes significant distress but can also trigger cardiovascular complications, POD, and delayed recovery [20]. Guided by ERAS principles, MMA has become a key strategy for optimizing perioperative care by integrating drugs with different mechanisms to achieve the core goal of “opioid sparing” [21]. This study prospectively evaluated the clinical value of paracetamol-mannitol injection as a preemptive measure in elderly THA patients. Paracetamol-mannitol injection is a novel intravenous analgesic. In this formulation, mannitol’s primary role is as a solvent and stabilizer, which increases the solubility and stability of the paracetamol, reduces the risk of crystallization, and ensures the safety of administration. It has been clinically proven to have a good central analgesic effect and an opioid-sparing effect. Its rapid onset of action following intravenous administration makes it suitable for early postoperative analgesia [22]. Our findings confirm that incorporating preemptive intravenous paracetamol-mannitol into an MMA framework effectively reduces early postoperative pain intensity, produces a significant.

opioid- sparing effect, and enhances patient comfort in elderly patients undergoing THA. Importantly, our study provides biomarker evidence suggesting that this interven- tion may improve early recovery quality by inhibiting parts of the surgery-induced inflammatory response and neuro-stress.

The present study used a single preemptive dose of intravenous paracetamol before surgical incision, which differed from several previous investigations that adopted postoperative or repeated dosing regimens. Compared with studies initiating analgesia after surgery, our preemptive approach was designed to suppress the early inflammatory and neurogenic stress responses triggered by surgical trauma at the initial stage. Although postoperative continuous administration can maintain stable analgesic effects and prolong drug exposure, our results suggest that the timing of administration may play a critical role in modulating early-phase biomarkers, including inflammatory factors and S100β. Therefore, the beneficial effects observed in this study may be attributed not only to the pharmacological properties of paracetamol itself but also to the preemptive timing of administration, which prevents central sensitization and excessive inflammatory cascade activation before noxious stimulation occurs.

### Significance of preemptive analgesia and opioid sparing

THA is associated with moderate-to-severe pain originating from extensive bone and soft tissue trauma [4]. In our study, VAS scores in the experimental group were significantly lower than in the control group at 6 and 24 hours postoperatively, the peak periods for pain intensity. This strongly supports the core concept of preemptive analgesia: intervening before the noxious stimulus occurs to prevent or reduce the establishment of central sensitization [23, 24]. Central sensitization, which manifests as hyperalgesia and allodynia, is a key mechanism underlying the persistence and intensification of postoperative pain. Although the mechanism of paracetamol is not yet fully elucidated, it is widely believed to act by inhibiting central cyclooxygenase (COX) isoenzymes, thereby reducing prostaglandin E2 (PGE2) synthesis [25]. Its metabolite, AM404, may also activate the endogenous cannabinoid system and TRPV1 channels [26]. By administering the drug preoperatively, we ensured that effective plasma concentrations were reached by the time of incision, thus ”blunting” the initial nociceptive signaling, reducing the excitability of the spinal dorsal horn and higher central nervous centers, increasing the pain threshold, and lowering postoperative pain intensity. This enhanced baseline analgesia translated directly into a significant ”opioid-sparing effect”: patients in Group A required less sufentanil, had fewer PCIA demands in the first 24 h, and had a significantly longer time to their first analgesic request. This result aligns perfectly with the primary goal of MMA, which is to use the synergistic effects of different analgesics to reduce reliance on opioids and their associated adverse effects [27]. For the physiologically vulnerable elderly population, minimizing opioid consumption is a key strategy to reduce the risk of postoperative nausea, vomiting, excessive sedation, and delirium, thereby facilitating early functional recovery.

### Modulation of inflammatory and neuro-stress responses

Surgery, as a controlled trauma, inevitably triggers the body’s stress and inflammatory responses, leading to the release of pro-inflammatory cytokines such as IL-1*β*, IL-6, and TNF-*α* [28]. Our study provides new evidence for the potential anti-inflammatory and putative neuroprotective effects of preemptive paracetamol-mannitol. Although postoperative IL-1*β* and IL-6 levels were significantly elevated in both groups due to surgical trauma, a significant difference was observed in the responses of TNF-*α* and S100*β* protein. The control group showed a significant postoperative increase in both markers, which may reflect a strong systemic inflammatory and neurological stress response. In contrast, this increase was effectively suppressed in the experimental group, with postoperative levels showing no significant difference from baseline. although paracetamol is rapidly metabolized and its plasma concentration decreases significantly within a few hours, its anti-inflammatory and neuroprotective effects may persist beyond its half-life. This may be related to the following potential mechanisms: ① Paracetamol can inhibit the synthesis of prostaglandins (a key mediator of inflammation) in peripheral tissues and the central nervous system, and this inhibitory effect may have a lasting impact on the acute inflammatory response induced by surgical trauma; ② Preemptive administration before surgical stimulation can block the “inflammatory cascade” at an early stage, thereby reducing the subsequent release of inflammatory markers (including TNF-α) and neuroprotective markers (S100β) within 24 h.Studies have indicated that intrathecal injection of pro-inflammatory cytokines such as TNF-α induces acute nociceptive behaviors in mice, while oral administration of paracetamol reduces such pain responses in a dose-dependent manner. Meanwhile, it has been further demonstrated that AM404, a downstream metabolite of paracetamol, can inhibit TNF gene transcription and protein synthesis, thereby revealing the molecular pathway underlying paracetamol-mediated modulation of TNF-α expression [29]. As a key upstream initiator of the inflammatory cascade, the attenuated TNF-α response observed in the experimental group suggests that paracetamol-mannitol may partially suppress the intensity of the systemic inflammatory response [30].

Particularly noteworthy is the stability of the S100*β* protein level. S100*β* is considered a biomarker for increased blood-brain barrier permeability and neuronal/glial cell injury, and its postoperative elevation is closely associated with an increased risk of postoperative cognitive dysfunction (POCD) and delirium in elderly patients [31]. These findings may suggest modulation of neuro-stress markers rather than definitive neuroprotection.This observation could be related to mitigating surgery-induced neuroinflammation, maintaining the integrity of the blood-brain barrier, or directly inhibiting central microglial overactivation, though these potential mechanisms remain speculative and suggest that this compound may possess putative neuroprotective properties that require further validation.

### Clinical safety and patient experience

In terms of safety, there were no statistically significant differences in the incidence of common adverse events like nausea and somnolence between the groups. Given the significantly lower opioid use in the experimental group, a corresponding reduction in opioid-related side effects would be theoretically expected. The failure to detect a difference may be due to the relatively small sample size, providing insufficient statistical power to detect differences in low-frequency events. Notably, there was a trend towards a lower incidence of postoperative delirium in the experimental group (1 case vs. 4 cases). Although not statistically significant, this finding is clinically suggestive. Continuous monitoring throughout the study demonstrated that hemodynamic parameters (systolic blood pressure, diastolic blood pressure, heart rate, and mean arterial pressure) were maintained more stably in Group A (acetaminophen mannitol group). This observation may be associated with the superior postoperative analgesic efficacy in this group—effective pain control can mitigate sympathetic nervous system activation induced by pain-related stress, thereby reducing the risk of hemodynamic fluctuations such as vasoconstriction and tachycardia. In contrast, patients in Group C exhibited a relatively higher amplitude of heart rate fluctuations, with the peak of these fluctuations showing a certain correlation with the time points of elevated Visual Analog Scale (VAS) scores.However, all hemodynamic fluctuations were within the clinically controllable range and could be rapidly corrected through emergency analgesic intervention and perioperative standard supportive care. No severe cardiovascular adverse events requiring special management were reported.Since severe pain, high opioid consumption, and systemic inflammation are all core risk factors for delirium, and our intervention targeted all three, this trend highlights the significant potential of this protocol in preventing POD in elderly patients, warranting further investigation in larger trials [32]. In contrast, the positive impact of the protocol on patient comfort was statistically significant at 6 h postoperatively. The BCS provides a more comprehensive assessment of a patient’s physiological and psychological well-being than the VAS score alone. Improving patient comfort during the difficult early postoperative period is crucial for promoting early ambulation and improving sleep quality, ultimately contributing to an accelerated overall recovery process [33].Regarding the safety profile concerning renal and hepatic function, the mannitol content in the formulation used (1.925 g) serves primarily as an excipient for stability and solubility. This dosage is substantially lower than the therapeutic threshold required for osmotic diuresis. Consequently, the risk of organ dysfunction or electrolyte imbalance is minimal in patients with normal baseline function [34, 35]. Our findings corroborate this, as routine laboratory monitoring revealed no clinical evidence of acute liver or renal injury (defined as a > 1.5-fold increase in ALT/AST or Creatinine) in either group.

### Limitations

This study has several limitations. First, the exclusion of patients with MoCA-BC ≤ may have reduced the representation of individuals at highest risk for postoperative delirium, limiting external validity; in addition, as a single-center study, the generalizability of our findings may be further restricted. Second, the study was underpowered to detect statistically significant differences in postoperative delirium incidence, and thus the observed numerical reduction should be interpreted cautiously. Finally, our follow-up was limited to 48 h postoperatively, which precluded an assessment of long-term functional recovery, chronic postoperative pain, or quality of life. Future multicenter, large-sample studies with long-term follow-up are warranted to further validate the long-term efficacy and underlying mechanisms

### Conclusion and outlook

In conclusion, a single preoperative intravenous infusion of paracetamol-mannitol injection, as part of an MMA strategy, can significantly reduce postoperative pain, achieve an opioid-sparing effect, improve early comfort, and potentially attenuate inflammatory and neuro-stress responses in elderly patients undergoing THA, with a good safety profile. These findings support its incorporation into routine clinical practice to optimize perioperative management and enhance the quality of early recovery in this vulnerable patient population.

## Supplementary Information

Supplementary Material 1.

Supplementary Material 2.

## Data Availability

The datasets analyzed during the current study are not publicly available but are available from the corresponding author on reasonable request.

## Abbreviations

THA: Total Hip Arthroplasty
PCIA: Patient-Controlled Intravenous Analgesia
VAS: Visual Analog Scale
BCS: Bruggemann Comfort Scale
POD: Postoperative Delirium
ERAS: Enhanced Recovery After Surgery
MMA: Multimodal Analgesia
WHO: World Health Organization
VCV: Volume-Controlled Ventilation
FiO_2_: Fraction of Inspired Oxygen
CAM: Confusion Assessment Method
PACU: Post-Anesthesia Care Unit
ELISA: Enzyme-Linked Immunosorbent Assay
ASA: American Society of Anesthesiologists
POCD: Postoperative Cognitive Dysfunction

## References

[1] Nayab S, Elahi MB. The impact of exercise interventions on pain, function, and quality of life in patients with osteoarthritis: a systematic review and meta-analysis. Cureus. 2024;16(11):e74464. 10.7759/cureus.74464 PMC11669877 39726491

[2] Kurosaka K, Tsukada S, Ogawa H, Nishino M, Nakayama T, Yoshiya S, Hirasawa N. Addition of corticosteroid to periarticular injections reduces postoperative pain following total hip arthroplasty under general anaesthesia: a double-blind randomized controlled trial. Bone Joint J. 2020;102(10):1297–302. 32993338 10.1302/0301-620X.102B10.BJJ-2020-0428.R1 PMC7517720

[3] Jin Z, Wang L, Qin J, Hu H, Wei Q. Direct anterior approach versus posterolateral approach for total hip arthroplasty in the treatment of femoral neck fractures in elderly patients: a meta-analysis and systematic review. Ann Med. 2023;55(1):1378–92. 37000019 10.1080/07853890.2023.2193424 PMC10071980

[4] Xia Q, Ding W, Lin C, Xia J, Xu Y, Jia M. Postoperative pain treatment with transmuscular quadratus lumborum block and fascia iliaca compartment block in patients undergoing total hip arthroplasty: a randomized controlled trial. BMC Anesthesiol. 2021;21(1):188. 34243719 10.1186/s12871-021-01413-7 PMC8272275

[5] Rivasi G, Menale S, Turrin G, Coscarelli A, Giordano A, Ungar A. The effects of pain and analgesic medications on blood pressure. Curr Hypertens Rep. 2022;24(10):385–94. 35704141 10.1007/s11906-022-01205-5 PMC9509303

[6] Sohn R, Assar T, Kaufhold I, Brenneis M, Braun S, Junker M, Zaucke F, Pongratz G, Jenei-Lanzl Z. Osteoarthritis patients exhibit an autonomic dysfunction with indirect sympathetic dominance. J Translational Med. 2024;22(1):467. 10.1186/s12967-024-05258-9 PMC11100157 38755685

[7] Ma J-H, Liu Y-F, Hong H, Li C-J, Cui F, Mu D-L, Wang D-X. Effect of acute pain on the association between preoperative cognitive impairment and postoperative delirium: a secondary analysis of three trials. Br J Anaesth. 2023;130(2):272–80. 35933172 10.1016/j.bja.2022.06.033

[8] Lan S, Liang S, Wu H, Deng S, Sun K, Ye C, Yang L, Ciren L, Li J. Strategies to prevent postoperative delirium: a comprehensive evaluation of anesthesia selection and drug intervention. Front Psychiatry. 2024;15:1518460. 39763689 10.3389/fpsyt.2024.1518460 PMC11701066

[9] Liu X-H, Zhang Q-F, Liu Y, Lu Q-W, Wu J-H, Gao X-H, Chen Z-Y. Risk factors associated with postoperative delirium in elderly patients undergoing hip surgery. Front Psychiatry. 2023;14:1288117. 37928911 10.3389/fpsyt.2023.1288117 PMC10620517

[10] Mossie A, Regasa T, Neme D, Awoke Z, Zemedkun A, Hailu S. Evidence-based guideline on management of postoperative delirium in older people for low resource setting: systematic review article. Int J Gen Med. 2022;15:4053–65. 10.2147/IJGM.S349232 PMC9014957 35444455

[11] Zhao P, Ying Z, Yuan C, Zhang H, Dong A, Tao J, Yi X, Yang M, Jin W, Tian W, et al. Shared genetic architecture highlights the bidirectional association between major depressive disorder and fracture risk. Gen psychiatry. 2024;37(3):101418. 10.1136/gpsych-2023-101418 PMC11086190 38737893

[12] McDaid C, Maund E, Rice S, Wright K, Jenkins B, Woolacott N, et al. Paracetamol and selective and non-selective non-steroidal anti-inflammatory drugs (nsaids) for the reduction of morphine-related side effects after major surgery: a systematic review. Health Technol Assess. 2010;14(17):1–153. 20346263 10.3310/hta14170

[13] L´eger M, Pessiot-Royer S, Perrault T, Parot-Schinkel E, Costerousse F, Rineau E, Lasocki S. The effect of opioid-free anesthesia protocol on the early quality of recovery after major surgery (sofa trial): study protocol for a prospective, monocentric, randomized, single-blinded trial. Trials. 2021;22(1):855. 34838109 10.1186/s13063-021-05829-x PMC8627013

[14] Vangala C, Niu J, Montez-Rath ME, Yan J, Navaneethan SD, Naik AD, Winkelmayer WC. Hip fracture risk among hemodialysis-dependent patients prescribed opioids and gabapentinoids. J Am Soc Nephrol. 2020;31(6):1325–34. 32371535 10.1681/ASN.2019090904 PMC7269355

[15] Chen L, He W, Liu X, Lv F, Li Y. Application of opioid-free general anesthesia for gynecological laparoscopic surgery under eras protocol: a non-inferiority randomized controlled trial. BMC Anesthesiol. 2023;23(1):34. 36707777 10.1186/s12871-023-01994-5 PMC9881250

[16] Vitola E, Buraka N, Erts R, Golubovska I, Miscuks A. Effect of different low doses of intrathecal morphine (0.1 and 0.2 mg) on pain and vital functions in patients undergoing total hip arthroplasty: a randomised controlled study. BMC Anesthesiol. 2022;22(1):377. 36471258 10.1186/s12871-022-01919-8 PMC9720955

[17] Freo U, Ruocco C, Valerio A, Scagnol I, Nisoli E. Paracetamol: a review of guideline recommendations. J Clin Med. 2021;10(15):3420. 34362203 10.3390/jcm10153420 PMC8347233

[18] Mallama M, Valencia A, Rijs K, Rietdijk W, Klimek M, Calvache JA. A systematic review and trial sequential analysis of intravenous vs. oral perioperative paracetamol. Anaesthesia. 2021;76(2):270–6. 32557588 10.1111/anae.15163 PMC7818191

[19] Fillingham YA, Hannon CP, Erens GA, Mullen K, Casambre F, Visvabharathy V, Hamilton WG, Della Valle CJ. The efficacy and safety of acetaminophen in total joint arthroplasty: systematic review and direct meta-analysis. J Arthroplast. 2020;35(10):2715–29. 10.1016/j.arth.2020.05.037 32563592

[20] Yang X, Li G-h, Wang H-j, Wang C-y. Continuous passive motion after total knee arthroplasty: a systematic review and meta-analysis of associated effects on clinical outcomes. Arch Phys Med Rehabil. 2019;100(9):1763–78. 30831093 10.1016/j.apmr.2019.02.001

[21] Chahar P, Agarwal D, Farag E. Evidence-based multimodal analgesia for peri- operative management of spinal instrumentation. Curr Anesthesiology Rep. 2018;8(3):298–305.

[22] Nam S, Yoo S, Park S-K, Kim J-T. Additive effect of a single intravenous dose of acetaminophen administered at the end of laparoscopic hysterectomy on postoperative pain control with nefopam and fentanyl-based patient-controlled analgesia: a double-blind, randomized controlled trial. BMC Anesthesiol. 2025;25(1):88. 39979845 10.1186/s12871-025-02971-w PMC11841248

[23] De Oliveira GS Jr, Castro-Alves LJ, McCarthy RJ. Single-dose systemic acetaminophen to prevent postoperative pain: a meta-analysis of randomized controlled trials. Clin J Pain. 2015;31(1):86–93. 25485955 10.1097/AJP.0000000000000081

[24] Shlaifer A, Sharfman ZT, Martin HD, Amar E, Kazum E, Warschawski Y, Paret M, Brill S, Drexler M, Rath E. Preemptive analgesia in hip arthroscopy: a randomized controlled trial of preemptive periacetabular or intraarticular bupivacaine in addition to postoperative intra-articular bupivacaine. Arthroscopy: J Arthroscopic Relat Surg. 2017;33(1):118–24. 10.1016/j.arthro.2016.07.026 27729164

[25] Kim SH, Kang H, Jun I-J, Park HW, Yoo BH, Lim Y-H, Kim K-M. Effect of perioperative intravenous ibuprofen versus acetaminophen on postoperative opioid consumption and pain after general anesthesia: a systematic review and meta-analysis with trial sequential analysis of randomized controlled trials. Korean J anesthesiology. 2024;77(4):455–67. 10.4097/kja.24089 PMC11294878 38711266

[26] Tsuzawa K, Onimaru H, Inagaki K, Izumizaki M. Involvement of cannabinoid receptors in depression of the putative nociceptive response in spinal cord preparations isolated from neonatal rats. J Physiological Sci. 2023;73(1):23. 10.1186/s12576-023-00881-5 PMC10717773 37803279

[27] Zhou Y, Yuan P, Xing Q, Jin W, Shi C. Efficacy of postoperative analgesia with intravenous paracetamol and mannitol injection, combined with thoracic paravertebral nerve block in post video-assisted thoracoscopic surgery pain: a prospective, randomized, double-blind controlled trial. BMC Anesthesiol. 2024;24(1):14. 38172686 10.1186/s12871-023-02386-5 PMC10765788

[28] Khosravi G, Mahmodi Khaledi M, Abedi A, Azad Chehr MJ, Heidari MM. Inflammatory factors before and after orthopedic surgery in patients with fractures following trauma. Archives Trauma Res. 2022;11(4):184–8.

[29] Hiroshi H, Matthew H, Hiroshi N et al. Systematic Review of Systemic and Neuraxial Effects of Acetaminophen in Preclinical Models of Nociceptive Processing. Journal of pain research. 2021;143521-3552. 10.2147/JPR.S308028. 10.2147/JPR.S308028 PMC8594782 34795520

[30] Dolev A, Yaari L, Kittani M, Yassin M, Gbaren M, Feicht E, Shemesh S, Haviv B. Efficacy of anti-inflammatory treatment versus rescue analgesia after arthroscopic partial meniscectomy in nonarthritic knees: A 3-arm controlled study. Orthop J Sports Med. 2021;9(3):2325967121991545. 33796593 10.1177/2325967121991545 PMC7983444

[31] Wi W, Kim H-J, Bang S, Kweon OJ, Kim D, Oh EJ. Effect of intravenous versus inhaled anesthetics on blood-brain barrier dysfunction and neuroinflammation in elderly patients undergoing major surgery: study protocol of a randomized controlled trial. Trials. 2024;25(1):684. 39415284 10.1186/s13063-024-08515-w PMC11481368

[32] Zheng M, Wang B, Mao M, Wu Y, Wang Z, Yang L. Intravenous acetaminophen for postoperative delirium in older patients recovering from major non-cardiac surgery: a randomised-controlled study protocol. BMJ open. 2025;15(5):097079. 10.1136/bmjopen-2024-097079 PMC12083319 40379334

[33] Tan P, Huo M, Zhou X, Zhao B. The safety and effectiveness of enhanced recovery after surgery (eras) in older patients undergoing orthopedic surgery: a systematic review and meta-analysis. Arch Orthop Trauma Surg. 2023;143(11):6535–45. 37389596 10.1007/s00402-023-04963-2

[34] Farahzad J, Fatemeh ZJ, Masomeh A et al. The Comparison of the Effect of Mannitol and N Acetyl Cysteine on Liver Function in Partial Hepatectomy. Anesthesiology and pain medicine. 2018;8(5):e79677. 10.5812/aapm.79677. 10.5812/aapm.79677 PMC6240830 30533390

[35] Cheriyan A, Mariappan R, Jeyaseelan L et al. Effect of intravenous mannitol on renal function in patients undergoing laparoscopic nephron-sparing surgery: a randomized, double-blinded trial..International urology and nephrology. 2025;57(12):1–8. 10.1007/S11255-025-04609-9. 10.1007/s11255-025-04609-9 40514624

