# Supplementary material for: The effect of preemptive intravenous paracetamol-mannitol on postoperative analgesia and quality of recovery in elderly patients undergoing total hip arthroplasty
Source: BMC Anesthesiol. 2026 Apr 9;26:314. doi: 10.1186/s12871-026-03815-x (PMC13188345; doi:10.1186/s12871-026-03815-x)
Supplement: Supplementary file 2 — Supplementary Material 2. [file 12871_2026_3815_MOESM2_ESM.docx]

**Figure 1: CONSORT 2025 Flow Diagram**

Analysis

Analysed for primary outcome (n= 33)

Excluded from analysis (give reasons) (n=0 )

Discontinued intervention (give reasons) (n=0 )

Lost to follow-up for primary outcome (give reasons) (n=0 ):

Discontinued intervention (give reasons) (n=0 )

Lost to follow-up for primary outcome (give reasons) (n=0 ):

Excluded (n= 16)

Not meeting inclusion criteria (n=10 )

Declined to participate (n=6 )

Other reasons (n=0 )

Randomised (n=66 )

Allocation

Follow-Up

Allocated to intervention (n=33 )

Received allocated intervention (n=33 )

Did not receive allocated intervention (give reasons) (n=0 )

Allocated to intervention (n=33 )

Received allocated intervention (n=33 )

Did not receive allocated intervention (give reasons) (n=0 )

Enrolment

Assessed for eligibility (n=82 )

Analysed for primary outcome (n= 33)

Excluded from analysis (give reasons) (n= 0)
